# Clinical Treatment Study of Secondary Multiple Squamous Cell Carcinoma with Psoriasis Vulgaris

**DOI:** 10.1155/2022/9529681

**Published:** 2022-01-06

**Authors:** Sai-Nan Han, Shi-Jun Feng, Dong-Fang Ai

**Affiliations:** Department of Dermatology, Cangzhou Central Hospital, Cangzhou, Hebei 061000, China

## Abstract

Dermatologic diseases are the fourth most frequent nonfatal common illness, yet they have a psychological, economical, and professional burden that is comparable to or larger than other chronic conditions. From a survey in China of 6 provinces, the overall prevalence of psoriasis with squamous cell carcinoma was 0.47%. According to the current investigation, the outburst of skin disease was not associated with gender, but mainly with the climate of the environment; that is, dry cold weather will more likely to induce psoriasis. Approximately 3% of people around the world have psoriasis, which is near the most common autoimmune skin disease in adults. By simple estimation, there are at least two hundred million psoriasis patients in the world. Therefore, it is not just a simple health problem in a country or a region but a serious global challenge. Of note, about half of the adult patients had been reported to be sick in their childhood and they mostly fell ill around 10 years old. Actinic keratosis is perhaps the most common, followed by squamous cell carcinoma and, to a lesser extent, acne vulgaris, psoriasis, and hidradenitis suppurativa, as well as dermatitis herpetiformis. 5-Fluorouracil (5-FU) 0.5 percent is used topically to treat actinic keratosis and squamous cell carcinoma with good outcomes, while it might cause significant toxicity in certain patients. Dapsone, a Valosin-containing protein, is a medication that is often used to treat inflammatory skin disorders like psoriatic arthritis, but it can occasionally cause hemolytic anemia. Furthermore, biologic medications for the treatment of moderate-to-severe psoriasis and multiple squamous cell carcinoma have proven to be successful and safe; nevertheless, a small percentage of patients do not react to biologic treatment in the long term or do not respond at all. Based on the data from the China Food and Drug Administration, the majority of chemical drugs are utilized as the topical formulations, while Chinese medicines are mainly delivered by an oral route, suggesting that the market for topical preparations of Chinese medicine to treat skin diseases like psoriasis is worth exploration. This large interindividual diversity in response could be caused by changes in genes that encode proteins implicated in the disease's pathologic environment or the medication's mechanism of action. Pharmacogenetics is the study of the association between genetic differences and medication response, which is valuable for identifying nonresponsive patients and those who are more likely to suffer toxicity as a result of treatment. This study highlights the pharmacogenetic recommendations for dermatology therapies that have the strongest evidence at this time, highlighting those that have been incorporated in clinical practice guides. Pharmacogenetic clinical guidelines for multiple squamous cell carcinoma and psoriasis vulgaris were found in this investigation. Here, for multiple squamous cell carcinoma trichohyalin-like 1 (TCHHL1), 5-fluorouracil (5-FU) 0.5% is recommended. Along with that dapsone, Valosin-containing protein can be recommended for treating the psoriasis vulgaris. We made some clinical trials over the two diseases, and from the result obtained, we hypothesize that the suggested drug may be a novel therapeutic target in treating the multiple squamous cell carcinoma with psoriasis vulgaris.

## 1. Introduction

Skin illnesses are one of the most common causes for seeking medical help, accounting for between 5.5 percent and 22.5 percent of all visits. Although most are not life-threatening, their emotional, economical, and occupational effect is thought to be comparable or greater than that of other chronic diseases; 25 percentage of dermatologic disorders, in particular, can result in impairment. Primary skin illnesses, cutaneous symptoms of systemic diseases, and sexually transmitted infections are the three types of skin illnesses like a genital herpes. Acne, skin irritation, warts, psoriasis, and vitiligo are the most frequent in Europe. Treatments for dermatological pathologies differ depending on the severity of the disease. The most common treatments are topically applied medications such as systemic corticosteroids and physiotherapy heating, freezing, lasers radiation, or electromagnetic waves. The most severe illnesses, on the other hand, necessitate the use of systemic medicines, biologic therapy, and surgical procedures. Although biological and systemic medication therapies are usually safe and effective, some patients need not react to them in the short term or long term and experience differing levels of toxicity. The requirement for prognostic and predictive indicators to guide treatment of skin treatment selection has led to the use of PGx pharmacogenetics [1]. This is a method that allows for the earlier diagnosis of patients who will receive treatment more and have a reduced likelihood of experiencing side events or toxicity. In the field of dermatological, pharmacogenetics development is currently on the rise. Large cohorts of patients with diverse of clinical characteristics have generated an immense number of genetic research in recent years. As a result, the EMA-European Medicines Agency and the United States FDA (Food Drug Administration) have included these genetic-based pharmacological treatment patterns into pharmacogenetics clinical guidelines or suggestions. However, because of the wide range of systemic medicines used in other diseases that might cause severe cutaneous symptoms, pharmacogenetics is especially important in dermatology. As a result, pharmacogenetics suggestions in dermatology are essential for avoiding cutaneous side effects and maximizing therapeutic effect. The healthcare system is currently facing a major problem in integrating pharmacogenetics into routine clinical practice.

**Figure 1:**
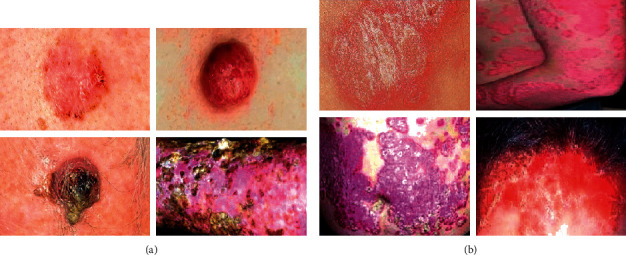
Sample images of multiple squamous cell carcinoma and psoriasis vulgaris. (a) Multiple squamous cell carcinoma. (b) Psoriasis vulgaris.

The application of pharmacogenetics in routine dermatologic clinical practice will be based on evaluation and analysis of pharmacogenetics studies, national healthcare organizations suggestions, and pharmacogenetics clinical guidelines, thereby increasing the patient's quality of life through individualized medication. The information is currently available on pharmacogenetically significant medications used to analyze skin conditions shown in Figure 1. Patients with actinic and basal cell carcinoma should use 5-fluorouracil topically, according to the pharmacogenetics clinical guidelines, whereas patients with dermatitis herpetiformis should use dapsone systemically, according to the pharmacogenetics clinical guidelines,. Psoriasis is more commonly found in psoriasis and SCC patients between the ages of 14 and 25. However, some of the early phases of this disorder may be confused with eczema or other inflammatory skin problems. There are two stages of SCC: in stage 1, the cancer has migrated deep into the skin but not to adjacent lymphatic system or normal tissues, and in stage 2, the cancer has gone deep into the skin and has one or more high-risk characteristics, such as metastases to neurons and lower skin levels, but it has not spread to the neighboring lymphatic system or normal tissues.

## 2. Related Work

TCHHL1 plays an important role in regular human keratinocytes (NHKs) and squamous cell carcinoma (SCC). NHKs were dramatically reduced in proliferation and triggered early apoptosis when TCHHL1 was knocked down by transfected with TCHHL1 siRNA. The phosphorylation of intracellular signal-regulated kinase 1/2 (ERK1/2) was dramatically reduced in TCHHL1-knockdown NHKs [2]. Furthermore, overexpression of forehead box-containing proteins O1(FOXO1), B-cell lymphoma 2 (BCL2), and Bcl2-like nutrient 11 (BCL2L11) was found, as well as a minor suppression of v-akt murine thymoma viral oncogene homolog (AKT) phosphorylation. TCHHL1-knockdown NHKs produced epidermis structures with a hyperplastic epidermal. [3] TCHHL1 is a new recruit of the fusion S100 family proteins. TCHHL1 has an EF-hand region, a gender fluid site, and a nucleotide binding signal, according to the determined amino acid. The production of TCHHL1 molecules in healthy and diseased skin cells was studied using a monoclonal antibody raised against the C-terminus of the TCHHL1 molecule. [4] The focus of this research was to see how ultraviolet B (UVB) irradiation affected TCHHL1 expression levels of skin xenotransplants. TCHHL1 expression levels increased by two days following UVB irradiation in UVB-exposed skins. Following sham irradiation, TCHHL1 was immunohistochemically found in the basal levels. [5] We discovered an ortholog of cornulin in birds and reptiles, as well as a previously undiscovered SFTP called scaffolding, but filaggrin was only found in mammalian. Cornulin and scaffolding are both produced in the developmental periderm of chickens, unlike mammal SFTPs. In terms of expression in the filiform papillae of the tongue and the epithelium beneath the developing tips of the claw, scaffolding parallels mammal trichohyalin. [6] Cornulin (CRNN) was immunohistochemically validated in a wider group of ESCC cases. Cornulin reduced expression was seen in 89 percent (*n* = 239) of the 266 ESCC tissues arrayed on tissue microarrays (TMAs). Cornulin genes are expressed in the prickly and cellular layers of the healthy mucous membrane, with the cytoplasmic and perinuclear area being the most prominent. [7] A total of 36 samples from a real-world cervical SCC cohort were examined. To identify various expression patterns of immune-related genes, we employed a nonnegative matrix factorization (NMF) technique (IRGs). We analyzed immunological features, putative immune biomarkers, and genomic instability. For verification, two separate datasets of 555 participants were employed. [8] Basal cell skin cancers account for about 80% of nonmelanoma skin malignancies, whereas squamous cell carcinomas account for 20%. (a) In white people, squamous cell carcinoma is the second most prevalent malignancy. (b) In contrast to practically all basal cell carcinomas, cutaneous squamous cell carcinomas have a significant risk of spreading. [9] Examining the cancer research gives the sense that data analysis for solutions to many crucial physiological, behavioral, prognosis, and treatment concerns about squamous cell carcinoma of the skin is challenging. The current thoughts, debates, and treatment for skin squamous cell carcinoma are discussed. [10] From 1988 to 1998, data of 201 individuals diagnosed with extensive SCC and handled by Mohs treatment were obtained from the Dermatologic Surgery Unit at the Medical University of Carolina's tumor database. A retroactive investigation was carried out. The chi-squared test and Miller's considered test were used to compare the features of patients with multiple myeloma SCC and those with nonmetastatic SCC. The current comprehensive evaluation included prospectively enrolled individuals with at least one psychopathically confirmed cutaneous SCC lesion, definitive treatment of the SCC lesion (s) resulting in no signs of disease, and at least 2 months of follow-up after therapeutic intervention. They were given complete medical and pathological examinations, as well as adopt for failures and high mortality [11, 12]. Following carcinoma, epidermal squamous cell carcinoma is the second most prevalent form of skin cancer, and also its prevalence is increasing. Squamous cell carcinoma biology is discussed in terms of genesis, immunobiology, biochemical, metastatic spread, and treatment, with a focus on prevention, diagnostics, and treatment [13]. The clinical symptoms of cSCC are used to make the diagnosis. In place to enable the prognosis categorization and proper care of cSCC, all clinically suspect lesions should undergo a biopsy or removal with histological verification. Thorough surgical removal with histological management of excised margins is the first-line therapy for cutaneous SCC. Including for low-risk tumors, the EDF–EADO–EORTC consensus committee advises a standardized minimum margin of 5 mm [14]. SCC's histology can be thought of as a spectrum of squamous intraepithelial neoplasia, with a number of common and atypical variations. Because invasive SCC has the ability to return and spread, it is critical to identify the characteristics that put specific lesions at a higher risk of recurrence or spread [12]. In the United States, nonmelanoma carcinoma is the most prevalent type of cancer. Cutaneous squamous cell carcinoma is the second most common type of skin cancer after basal cell carcinoma, and its frequency is rising [3]. Squamous cell biology is discussed in terms of genesis, immunobiology, metabolism, metastatic spread, and treatment, with a focus on prevention, diagnostics, and treatment [15, 16]. Patients with nail unit SCC who had Mohs micrographic surgery for their cancer were studied to better understand the clinical features and diagnostic factors involved [16]. Use the EudraVigilance reporting system to evaluate the incidence of psoriasis as a particular immune-related cutaneous adverse event linked to ICIs.

## 3. Materials and Methods

### 3.1. Trichohyalin-like 1 (TCHHL1) for Squamous Cell Carcinoma

**Figure 2:**
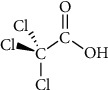
Chemical structure for TCHHL1.

Epidermal growth is a challenging process that needs the coordinated and progressive expression of a number of genes. Many genes implicated in this condition are found in the epidermis developmental region, a 2 Mb area on chromosome bands 1q 21.3. This region includes “fusion S100 molecules,” which have an EF-hand domain at the N-terminus followed by multiple sequential peptide repeats. The bulk of fusion S100 molecules, such as filaggrin, are expressed in the granular layer, and all these proteins are assumed to be involved in barriers or cornification formation. This theory is supported by the findings that a deletion in the filaggrin gene causes atopic psoriasis or ichthyosis vulgar, as well as a decrease in the development of filaggrin-like protein in atopic skin. TCHHL1 is a newly discovered component of the fusion S100 family proteins AY456639. We have considered 1456 for analysis of this study which was under treatment in Huashan Hospital, and it is quite an access code in GenBank. The human TCHHL1 gene encodes a 904-amino-acid protein with an EF-hand region at the N-terminus following a big region, according to the determined amino acid composition (Figure 2).

**Figure 3:**
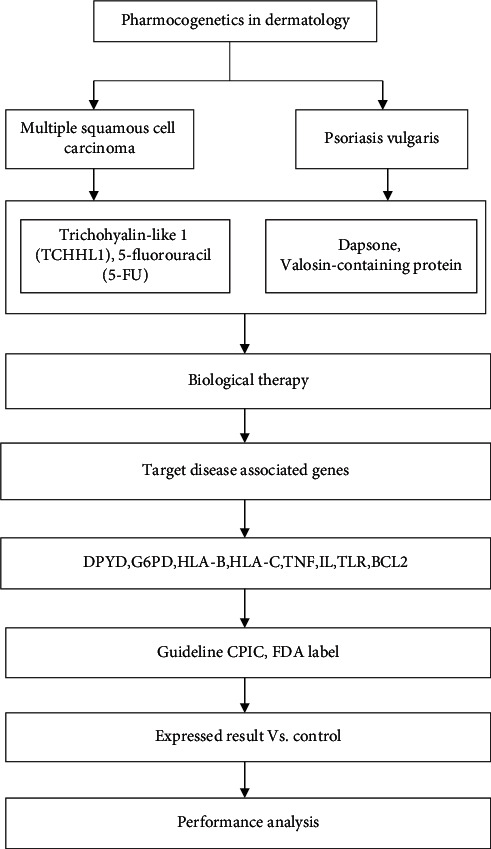
Schematic representation of the suggested methodology.

The whole experimentation was carried out in a XiangyaDerm China database. It contains 107,565 clinical images, covering 541 types of skin diseases. Each image in this dataset is labeled by professional doctors. As far as we know, this dataset is the largest clinical image dataset of Asian skin diseases used in the computer-aided diagnosis (CAD) system worldwide. At the healthy epidermal, TCHHL1 is produced in the basal cell layer. TCHHL1 transcription was shown to be higher in samples obtained with keratinocyte hyperproliferation, including in psoriatic arthritis, squamous cell carcinoma, and basal cell carcinoma. TCHHL1 protein was also increased in developing keratinocytes of the epidermal that had been injured by UV irradiation. TCHHL1 appears to be linked to keratinocyte proliferation, according to these data. The significance of TCHHL1 in normal human keratinocytes (NHKs) versus SCC cells was explored in this work. As a consequence, the findings of this study revealed that TCHHL1 is linked to NHK and SCC cell growth and anti-apoptosis through AKT or ERK1/2 activation and that TCHHL1 thus plays a crucial role in normal epidermal and dermatological SCC equilibrium (Figure 3).

### 3.2. 5-FU (5-Fluorouracil)

**Figure 4:**
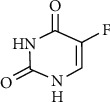
Chemical structure for 5-FU.

5-Fluorouracil (5-FU) is an intramuscularly given purine analog, which is used in the treatment of basal-cell carcinoma and actinic keratosis dermatologically at a low dose (0.5%) and in combination with salicylic acid (to boost the drug's absorption through the epidermis and contributing to its keratolytic impact). This drug is a pyrimidine analog that interacts with DNA and RNA production by irreversibly inhibiting the enzyme that catalyzes synthase, which prevents the transfer of deoxyuridilic acid to thymidylic acid. It also causes changes in the complex that controls RNA breakdown, and it impacts organisms with an established norms rate more frequently, resulting in toxic and cellular damage (Figure 4).

It is processed by the enzymes and uses a structure of dehydrogenase (DPD), which produces dihydrofluorouracil, an inactive metabolite that regulates the 5-FU plasma level. Because 85 percent of the amount supplied is swiftly removed, DPD's activity is critical in assessing the patient's therapeutic efficacy. The DPYD gene on chromosomal 1p 21.4 encodes the DPD enzymatic, and mutations in this gene cause a decrease in enzyme activity, leading to an increase in the half-life of 5-FU and, as a result, a higher risk of adverse effects. Around 3–5% of people have a mutation in the DPYD gene that causes a deficiency in this enzyme, putting them at an increased risk of toxicity while using 5-FU. Several DPYD genetic variations have been found, the most prominent of which are rs3918290 (G > A), rs55886062 (T > G), rs67376798 (A > T), and rs75017182 (C > G), which result in a considerable reduction in enzymatic activity and an increased risk of toxicity. The variations rs3918290 and rs55886062 have the most negative effects on DPD function, whereas rs67376798 and rs75017182 have such a moderate effect.

### 3.3. Dapsone

**Figure 5:**
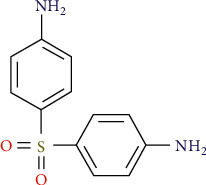
Chemical structure for dapsone.

Dapsone can be taken orally or used topically. Medical studies have shown that oral dapsone medication causes dose range-related hemolytic and hemolysis anemia and that those studies with the G6PD enzyme inhibitor are more susceptible. Despite the fact that there are presently no PGx prescriptions for this medicine in treatment guidelines, qualified healthcare authorities in several countries, like the FDA, have placed cautions in the description of product attributes for external dapsone (Figure 5).

They emphasize that precautionary measures must be taken during administering drugs, stating that susceptibility is higher based on the G6PD genetic, especially when dapsone is combined with some other medicines or given at massive doses, and also that hemoglobin levels in such patients must be monitored closely during diagnosis. Even though the number of alleles of these genetic changes is limited in European, the concentrations of these enzymes are measured in all individuals as part of a procedure prior to the introduction of certain oxidizing agents like dapsone. Nevertheless, no genotyping of the G6PD enzyme has already been carried out to establish whether genotype (B/A+/A−) is most important for preventing oxidizing drug side effects.

### 3.4. Valosin-Containing Protein

Psoriasis is a recurrent, chronic inflammatory skin condition that affects 2% of Caucasians but only 0.4 percent of the Japanese population. In systemic inflammation, aberrant growth of keratinocytes and infiltration of immune cells, mostly T cells and dendritic cells, are histologically identified. Psoriasis biomarkers were used to assess clinical symptoms, objectively track treatment response, discover new therapeutic targets, and explain comorbidity in patients with psoriasis. Nevertheless, no illness or diagnosis serum biomarker for psoriasis vulgaris has yet been discovered. The hunt for new psoriasis biomarkers is critical not just for developing diagnostic tools, but also for developing new therapies. We originally described psoriasis-associated protein molecules that have been recognized by auto-antibodies in serum samples from patient populations with psoriasis vulgaris for first immune response by immunoblotting predicated on two-dimensional gel electrophoresis to discover and difference pharmacomarkers of psoriasis vulgaris. They discovered autoantigens are Valosin-containing protein (VCP) in sera from psoriasis vulgaris as a result of our research. Serum from psoriasis vulgaris sufferers had considerably greater concentrations of moesin and STIP1 than control subjects. Furthermore, stress-induced phosphoprotein-1 was considerably higher in psoriatic arthritis sufferers' serum than in psoriasis vulgaris clients' serum. Strain phosphoprotein-1 and moesin differentiated sero-diagnostic markers of psoriatic arthritis, according to previous research. Nevertheless, a comprehensive research of serum VCP protein intake in patients with psoriasis has yet to be carried out. Comparative analysis with control subjects, we investigated whether the blood VCP protein level was a valuable diagnostic marker in patients with psoriasis vulgaris.

## 4. Types of Gene

### 4.1. Glucose-6-Phosphate Dehydrogenase (G6PD)

The G6PD gene produces the cytosolic enzyme G6PD, which metabolizes the molecule of glucose, converts it to 6-phosphogluconolactone, and produces NADPH; it is the first step in the polyol pathway. Because this enzyme is mostly generated in erythrocytes, it is one of the primary sources of NADPH in erythrocytes. G6PD is a polymorphic gene, with much more than 400 single-nucleotide polymorphisms identified, 186 of which are linked to decreased G6PD enzymatic stability and activity. Polymorphisms in the gene induce G6PD deficiency, which shows clinically anemia, are (a) acute hemolytic anemia, (b) chronic hemolytic, and (c) neonatal jaundice; however, most patients with this genetic disorder remain asymptomatic. Because the G6PD gene is on chromosomal X, genetic changes are passed down through the generations in a sex-linked recessive manner; that is, men are classified into G6PD normal or deficient, whereas ladies are split into 3 G6PD cell types: deficient, normal, intermediate. G6PD deficiency affects and over 400 million people globally, the highest incidence of human enzymopathy. As a result, enzymatic reaction assays are used to phenotypically identify G6PD insufficiency. Patients with normal enzyme activity, on the other hand, experience hematopoietic toxicity in the same way. This variation in dapsone toxicity across individuals could be attributable to hereditary factors. The polymorphisms that diminish G6PD activity have been investigated the most, and they are more common among Africans, especially in disease areas. As a result, the majority of investigations have been undertaken on individuals of this sort. Research with 117 patients and 23 controls in a sub-Saharan African population investigated the triallelic G6PD gene in particular. The G6PD-type B allele, which has normal enzyme activity, is the most prevalent variety worldwide. The G6PD group A+ allele, rs1050829, A376G (Asn Asp), preserves 85 percent of enzyme activity and is thus moderately deficient, whereas the G6PD-type A− allele, rs1050828 A376G and G202A (Val Met), decreases enzymatic activity to 12 percentage points and is thus seriously lacking. G6PD-type A− deficiency accounts for 90% of all G6PD deficiencies in tropical Africa. Dapsone, on either hand, can be applied topically to the skin in low quantities to treat acne vulgaris. The danger of hemolytic and/or hemolytic anemia in participants with G6PD enzyme inhibitor was investigated in a study including 56 Caucasian patients from the United States who were identified with acne vulgaris and treatment with topical dapsone. Despite the fact that respondents of this trial demonstrated that low-dose topical dapsone did not pose a clinically relevant risk of hemolysis or anemia in these patients, the FDA advises against using this medicine in patients with G6PD deficiency.

### 4.2. HLA Gene

Histocompatibility complex (HLA-B) belongs to the class I heavy chain paralogues. The HLA-B gene is part of the major histocompatibility group and is found on chromosomes 6p21.33 (MHC). One of human leukocyte antigens, HLA-B, connects with NK and T cells and their receptors in specific. Dapsone attaches to the human leukocyte antigen protein's Ile95 residue, changing the antigen identification site's architecture. Scars are formed when the body recognizes its own ligands as foreign agents. In the 4–6 weeks after starting dapsone therapy, this hypersensitive reaction typically manifests as fever, skin disease, and major organ damage. There has been no research on the HLA − B*∗*13 : 01 haplotype's effect on DH. In research of 872 Asian leprosy-treated patients with dapsone, the existence of the HLA − B*∗*13 : 01 allele was found to be an effective risk factor for dapsone hypersensitive syndrome with 85.5 percent sensitivities and an 85.7 percent specificity. Patients with 2 copies of the gene have a higher risk of hypersensitivity reactions than patients with a single allele, by these findings. Those that do not have a complement of the gene, on the other hand, have a really low or nonexistent chance of scars. To lessen the chance of developing hypersensitive responses, this study proposes omitting dapsone from treatment in individuals who have one or even more alleles of the HLA − B*∗*13 : 01 allele. The allelic variants of the HLA gene have already been extensively investigated in PGx, with inconsistent results. HLA molecules, which are part of the MHC, help the immune system recognize protein molecules that might trigger an antibody response. HLA system, which is found on chromosome at the PSORS1 gene, produces a wide range of HLA proteins with different functions. Anti-TNF medication responsiveness has been associated with HLA − Cw*∗*06 or ins haplotypes or HLA-A or LCE 3C 3B del or TRAF3IP2 rs13190932 haplotypes, and also HLA-C rs610604 polymorphisms.

### 4.3. TNF

Growth factors, receptors, and other related proteins, which are targets of biologic medicines, play a part in the growth and management of psoriasis. As a result, changes in the genes that code for those proteins are linked directly to medication reactions. The tumor necrosis factor (TNF) gene has over 200 variations, four of which were intensively investigated in psoriasis due to their relevance to the disease's physiopathology. The TNF is also a target for several biologics are CTL, ADA, INF that are used in treating modest inflammation. As a result, genetic changes in TNF could affect how these medications work. TNF-857, 308, 238 (rs1799724, rs1800629, rs361525 respectively) polymorphisms have been linked to psoriatic susceptibility and anti-TNF responses in psoriasis and other inflammatory illnesses like Crohn's disease and ankylosing spondylitis. Furthermore, the TNF-1031 (rs1799964) polymorphisms, which are found in the TNF gene's regulatory regions, have been linked to anti-TNF responses. TNF alpha-induced protein 3 (TNFAIP3) gene mutations have also been researched extensively in numerous inflammatory diseases. Nevertheless, only two polymorphisms, rs6920220 and rs610604 TNFAIP3, have been investigated in psoriasis patients treated with UTK and anti-TNFs, with inconsistent outcomes Types TNF binds to two types of receptors: TNFR1-TNF receptor type 1 (CD120a; p55/60) and TNFR2-TNF receptor type 2 (CD120b; p75/80). TNFR1 has a molecular weight of 55 kDa, whereas TNFR2 has a molecular weight of 75 kDa.

### 4.4. TLR

TLRs-Toll-like receptors are integral membrane proteins that play an important function in the immune system. TLR2 (rs4696480 and rs11938228), TLR5 (rs5744174) and TLR9 (rs352139) polymorphisms were found to affect treatment response in Caucasian individuals with chronic psoriatic (*n* = 376) and UTK (*n* = 230) who were medicated using anti-TNF medicines (*n* = 376) and UTK (*n* = 230).

### 4.5. Hidradenitis Suppurativa

To avoid serious complications, treating this disease is a major issue. Because of the positive outcomes seen in phase III clinical trials, the use of ADA and INF has just been allowed in individuals who do not respond to systemic medications or for whom they are inappropriate (PIONER I and II). Many PGx studies have been conducted to assess the efficacy of biologic therapy in diverse diseases. Only two trials in patients with mild to moderate HS have been undertaken in the case of HS. The researchers looked examined the effect of different polymorphisms in the TNF and TLR4 genes on anti-TNF medication therapeutic efficacy in 190 Caucasian patients with HS (from Greece), 32 of which were medicated with ADA. TNF-238 rs361525, a polymorphism in the promoter region of the TNF gene, was found to be linked to illness susceptibility and severity. Nevertheless, there was no statistically meaningful link between such anti-TNF and polymorphisms medication response (*p* > 0.05). Following that, in the United States, a genome-wide association analysis was done in individuals diagnosed with HS with the goal of finding the genetic variations linked to a reaction to ADA (*n* = 307). Attributed to the reason that the BCL2 gene encodes a regulatory protein of TNF suppression in follicle cells, the rs59532114 polymorphisms of the BCL2 genes were related to a poorer reaction to ADA. In furthermore, 269 Caucasian patients with HS and 365 with rheumatoid arthritis treated with ADA were evaluated to see if HLA type was linked to a lack of response to ADA. Only two HLA-DRB1 variations were discovered to be linked to an increased incidence of antidrug antibodies, which can lead to antimicrobial therapy.

### 4.6. Psoriasis

Psoriasis is a recurring inflammatory disease that causes up to 8.49 percent of adults and 2.1 percent of kids globally. Except in rare cases of erythema or pustular psoriatic, the cutaneous symptoms are not life-threatening. However, it has a negative influence on the patients' quality of life and results in excessive medical costs. It is also connected with other illnesses; hence it is considered a systemic phenomenon rather than a strictly dermatologic disease. Its cause is unknown, even though it is believed to be a result of genetics, environment, and immunologic variables (stress, traumas, medications, and microbial diseases are only a few examples). It has been discovered that the prevalence varies by ethnicity, is higher among relatives, and is considerably higher among monozygotic. Due to its regulatory function in autoimmunity against melanocytes, the presence of the HLA − C*∗*06 : 02 allele has been described as a factor in psoriasis susceptibility. Changes in cutaneous immune responses, mediated by dendritic cells activated by Toll-like receptors, produce a cascade of cytokines (TNF, IL-17, IL-23, and IL-12), which trigger the hyperproliferation of keratinocytes in the epidermis and give rise to the appearance of epidermal hyperplasia typical of psoriasis, which cause psoriatic lesions. On the scalp, elbows, knees, and back, 90% of patients develop erythematous plaques coated in whitish scales. Psoriasis area severity index (PASI), body surface area (BSA), and dermatology life quality index (DLQI) indicators are used to determine the severity of the lesions. Psoriasis with a PASI > 10, BSA > 10, and DLQI > 10 is termed moderate-to-severe psoriasis. Absolute PASI readings or percentage improvements in PASI are used to assess therapy efficacy; for example, a 90% reduction in lesions is considered effective (PASI90). Topicals, phototherapy, traditional systemic immunomodulators, or biologic therapy may be utilized to block the inflammatory response, depending on the severity of the psoriasis. Systematic treatment photochemotherapy or phototherapy are suggested in situations of moderate-to-severe psoriasis, and biologic therapy is used as a last resort when earlier therapies have failed or are contraindicated.

## 5. Results and Discussion

The most current PGx findings in the treatment of dermatologic illnesses. It is separated into four tiers depending on the kind of treatment: TCHHL1 and 5-FU are recommended for squamous cell carcinoma, whereas dapsone and Valosin-containing protein are recommended for psoriasis vulgaris. Finally, Table 1 depicts biologic therapeutic therapy.

### 5.1. TCHHL1 Cell Growth

**Figure 6:**
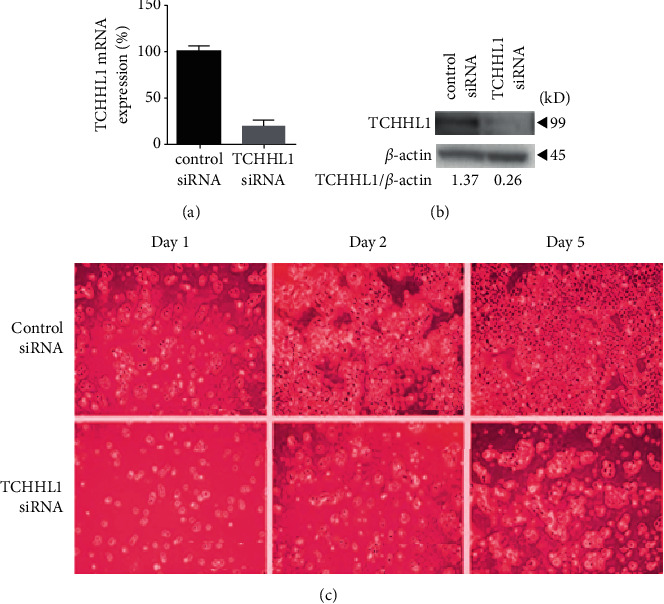
TCHHL1 cell growth level.

TCHHL1 siRNA was used in a siRNA experiment to see how TCHHL1 affects NHKs. TCHHL1 transcription factor and mRNA levels were lowered by TCHHL1 (s43057) treatment to 17.1% and 19.0% of control levels, respectively, as shown in Figures 6(a) and 6(b).

NHKs treated with TCHHL1 siRNAs had their vitality measured 1, 3, and 5 days after transfection. TCHHL1-knockdown NHKs showed a considerably lower number of viable cells 1 day after transfection than control NHKs (Figure 6(c)).

**Figure 7:**
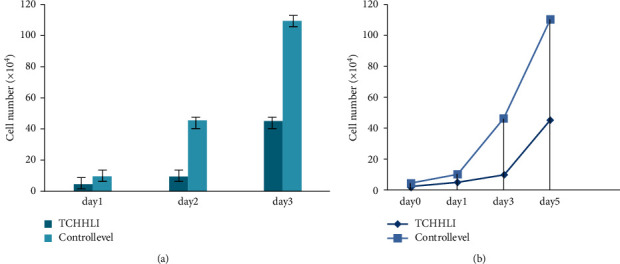
(a, b) Day by day, the problem level is controlled by TCHHL1.

Figures 7(a) and 7(b) depict the progression of the multiple squamous cell carcinoma skin illness over time with the use of the TCHHL1 medication.

### 5.2. 5-FU Cell Growth

**Figure 8:**
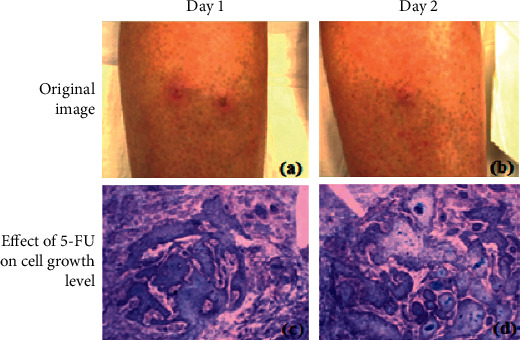
5-FU cell growth level.

Figure 8 shows the 5-FU medicine's growth level, which aids the drug's absorption through the epidermis and has a keratolytic impact. Keratolytics are substances that tear down the outer layer of the skin and aid in the retention of water. Acne, warts, and psoriasis are among the skin problems they address. Nevertheless, the aggressiveness of squamous cell carcinoma of the skin is typically not fatal. If left untreated, squamous cell carcinoma of the skin may spread to other regions of your body, resulting in major health issues.

**Figure 9:**
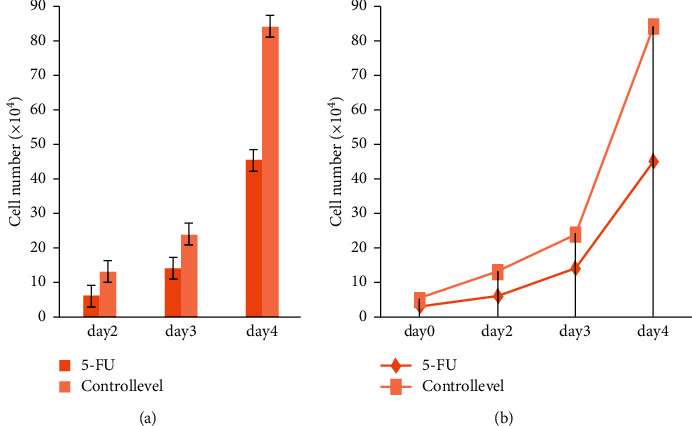
(a, b) Day by day, the problem level is controlled by 5-FU.

Figure 9 shows day-by-day improvement with the use of the 5-FU medicine in treating the multiple squamous cell carcinoma skin illness.

### 5.3. Dapsone Cell Growth

**Figure 10:**
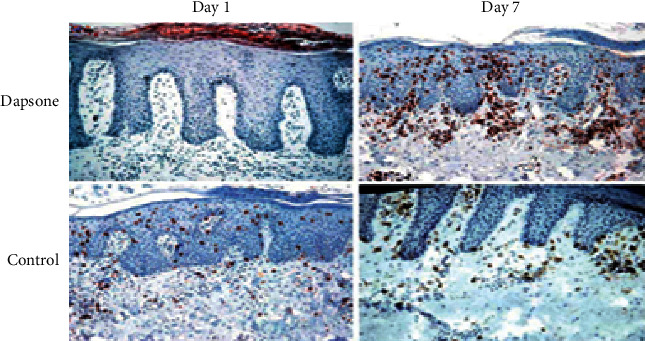
Dapsone cell growth level.

A medical study shows that oral dapsone treatment causes dose-related hemolytic and hemolysis anemia cell proliferation (see Figure 10).

**Figure 11:**
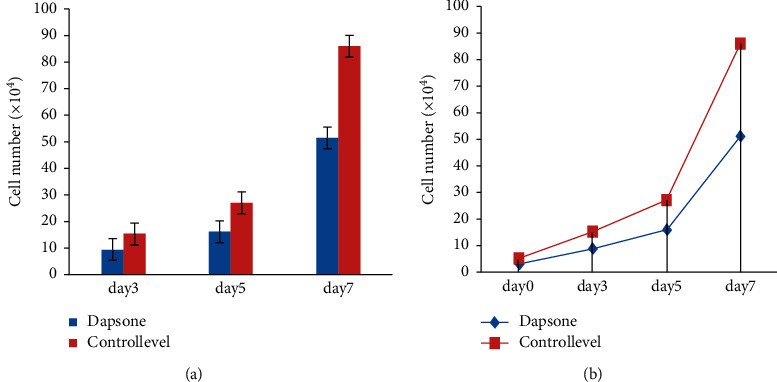
(a, b) Day by day, the problem level is controlled by dapsone.

Figure 11 shows day-by-day improvement with the use of the dapsone medicine in healing the psoriasis vulgaris skin illness.

### 5.4. Valosin-Containing Protein

**Figure 12:**
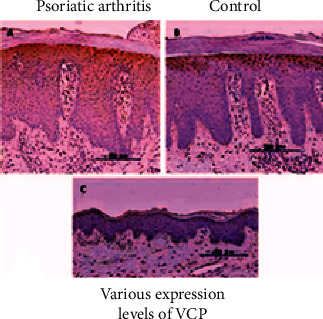
Valosin-containing protein cell growth. (a) Psoriatic arthritis. (b) Control. (c) Various expression levels of VCP.

**Figure 13:**
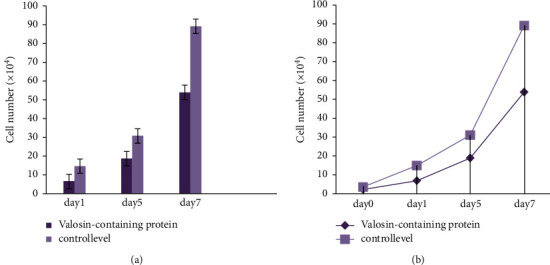
(a, b) Valosin-containing protein regulates the problem level on a daily basis.

VCP activity was detected in the cytoplasmic of the top stratum granulosum and the basal level of the epidermal of 38 of 42 psoriatic skin samples, including psoriasis vulgaris (Figures 12(a) and 12(b)), but not in 16 standards (Figure 12(c)). Despite excellent transcription, it was greater in psoriasis epidermal patients than in responders (*P* 0.01). There was no clear link between such protein expression profiles or locations and clinic pathological parameters such as gender, age, psoriasis vulgaris, BASDAI, DAS28, PASI scores, or the BSA involved component.  Figure 13 shows day-by-day improvement with the use of the dapsone medicine in healing the psoriasis vulgaris skin illness.

## 6. Conclusion

Finally, the adoption of PGx in dermatology has been slower than in other medical fields, such as cancer. The treatments for the illnesses listed above have been the most thoroughly researched, with considerable PGx findings. In this article, we look at different medicine types for two different skin conditions. Furthermore, biologic therapy suggested for diseases such as HS or moderate-to-severe psoriasis has shown promising outcomes; however, some patients do not achieve the intended benefit in the long term or short term, and experience varying degrees of toxicity. Many research has looked into the impact of polymorphisms in genes involved in the disease's pathological surroundings, metabolic, or mode of action on the efficacy of these drugs. Nevertheless, PharmGKB has a poor degree of evidence, and more validating studies are needed before this knowledge can be used in clinical practice in order to reduce the formation of cutaneous adverse events; it is equally critical to transfer PGx guidelines in other systemic medications for practical purpose.

## Figures and Tables

**Table 1:** Gene variants that affect dermatology response to therapy results for 5-FU and dapsone.

Gene	Polymorphism	Drug	Population	Level of evidence	Application in clinic	Category
HLA-B	13 : 01 : 01 HLA − B^*∗*^	Dapsone	Africans	1B	No	Toxicity
G6PD	rs1050829 A−	Dapsone	Asian	2A	No	Toxicity
	rs1050830 A+	Dapsone	Africans	—	No	Toxicity

DYPD	rs67376798	5-FU	Several groups	1A	Yes	Toxicity
	rs3918290	5-FU		1A	Yes	Toxicity
	rs55886062	5-FU		1A	Yes	Toxicity

## Data Availability

The data used to support the findings of this study are available from the corresponding author upon request.
